# Correlation between scalp high‐frequency oscillations and prognosis in patients with benign epilepsy of childhood with centrotemporal spikes

**DOI:** 10.1111/cns.14246

**Published:** 2023-05-08

**Authors:** Yichen Ji, Jun Zhang, Hongjuan Lu, Haoran Yang, Xuan Zhang, Huixin Liu, Wenjian Liu, Wei Zhou, Xiaoling Zhang, Wei Sun

**Affiliations:** ^1^ Department of Neurology Xuanwu Hospital Capital Medical University Beijing China; ^2^ Department of Neurology Mine Hospital Xuzhou China

**Keywords:** atypical forms of benign epilepsy of childhood with centrotemporal spikes, benign epilepsy of childhood with centrotemporal spikes, high‐frequency oscillations, prognosis

## Abstract

**Aims:**

The study aimed to explore whether high‐frequency oscillations (HFOs) can predict seizure risk and atypical manifestations of benign epilepsy of childhood with centrotemporal spikes (BECTS).

**Methods:**

We recruited 60 patients and divided them into three groups: (1) seizure‐free BECTS, (2) active typical BECTS, and (3) active atypical forms of BECTS. Electroencephalogram was used to record the number, location, average amplitude, and duration of spikes, and spike ripples were analyzed using time‐frequency technology. Multivariable logistic regression analysis was used to investigate independent predictive factors for prognosis.

**Results:**

The number of sleep spike ripples, rather than spikes, was an independent risk factor for the active period of the disease (odds ratio [OR] = 4.714, *p* = 0.003) and atypical forms of BECTS (OR = 1.455, *p* = 0.049); the optimal thresholds for the spike ripple rate were >0 (area under the curve [AUC] = 0.885, sensitivity = 96.15%, specificity = 73.33%) and >0.6/min (AUC = 0.936, sensitivity = 84.21%, specificity = 96.15%), respectively. Furthermore, in typical BECTS, the spike ripple rate showed significant negative correlations with time since the last seizure (*ρ* = −0.409, *p* = 0.009) and age (*ρ* = −0.379, *p* = 0.016), while the spike rate did not.

**Conclusion:**

Spike ripple was a marker for distinguishing typical and atypical forms of BECTS and reflected the risk of seizure recurrence better than the spike alone. The present findings might assist clinicians in BECTS treatment.

## Graphical abstract

More than 0.6/min of spike ripple rate can predict for atypical forms of benign epilepsy of childhood with centrotemporal spikes (BECTS), and the presence of spike ripple is an indicator for an active period of the disease, whereas the spike is insufficient to be an independent predictor. The spike ripple is more closely related to epileptogenesis than the spike alone.

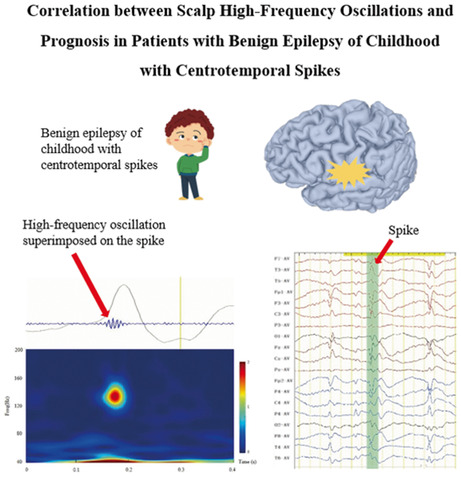

## 1 INTRODUCTION

Benign epilepsy of childhood with centrotemporal spikes (BECTS) is the most common childhood epilepsy syndrome remitting in late adolescence.^1^, ^2^ Electroencephalogram (EEG) shows high‐amplitude centrotemporal spike waves activated during sleep^2^ in patients with BECTS. Seizure frequency is usually low, but some children suffer from frequent seizures and require continuous treatment.^3^ A minority of patients might have common manifestations and EEG features at the early stage but more severe seizures and neuropsychological impairment later, which are called the atypical forms of BECTS.^4^, ^5^

Previous investigations on the prognosis of BECTS focused more on spikes, but the prediction accuracy of spikes was low.^6^ High‐frequency oscillation (HFO) is an EEG indicator that mainly consists of ripples (80–250 Hz) and fast ripples (250–500 Hz), which are more closely related to epileptogenesis than spikes.^7^, ^8^, ^9^ HFOs were first recorded with a microelectrode^10^ and then with an invasive intracranial electrode clinically.^11^ In 2010, Kobayashi et al.^12^ originally described HFOs in scalp EEG of continuous spike‐waves during slow‐wave sleep (CSWS). We also previously reported HFOs in eight patients with BECTS in a cognition‐related study.^13^ Notably, scalp ripples have been confirmed to be related to epileptic activity in children with West syndrome^14^ and respond to antiepileptic drugs (AEDs) better than spikes in BECTS.^15^ More ripples might indicate more frequent seizures in patients with rolandic spikes.^16^, ^17^ Moreover, accumulating evidence indicates that scalp‐recorded HFOs are more prominent in atypical and symptomatic rolandic epilepsy and CSWS than in typical BECTS,^8^, ^15^, ^18^ and ripples co‐occurring with spikes have greater pathological significance than ripples alone.^16^, ^17^, ^18^

A few studies have identified scalp‐recorded HFO as a new biomarker; however, these studies have several limitations due to sample capacity and some clinical confounding factors.^8^, ^16^, ^17^, ^18^ Moreover, there is debate about its cut‐off value for distinguishing between typical and atypical forms of BECTS and estimating disease activity.^16^, ^17^ Accordingly, this study aimed to evaluate the relationship of spikes and spike ripples with the outcomes of BECTS and identify EEG biomarkers to predict seizure risk and atypical manifestations.

## 2 METHODS

### 2.1 Patients

We recruited 60 patients who visited the Department of Neurology, Xuanwu Hospital, Capital Medical University, between July 2018 and August 2022. We reviewed the following clinical information: the age at seizure onset, seizure types, seizure frequencies, time since the last seizure, and medication conditions. Based on the clinical manifestations, we divided these children into three groups: (1) typical seizure‐free BECTS, (2) typical BECTS with active disease, and (3) active atypical forms of BECTS.

The diagnosis of typical BECTS followed the 1989 ILAE criteria: (1) focal seizures, which may be secondary to the general tonic–clonic seizures; (2) seizures usually occurring during sleep; (3) interictal EEG showing normal background and centrotemporal spike waves activated by sleep; and (4) normal neurological examination. The following criteria were used to define the patients with atypical features^1^: (1) early onset of typical BECTS and transition to more serious seizure forms, such as negative myoclonus and atypical absences; (2) transient oromotor dysfunction, such as hypersalivation or difficulties in articulation and pronunciation; (3) EEG showing a spike–wave index ≥50% during non‐rapid eye movement (NREM) sleep, and the patients had cognitive abnormalities. Children with other severe systemic diseases or magnetic resonance imaging abnormalities were excluded from this study. Patients with active disease were defined as having had at least one seizure within the last 1 year, while seizure‐free patients had no seizure for at least 1 year, as described previously.^17^ Twelve months of seizure freedom indicates a low risk of recurrence in the majority of children with epilepsy.^19^

The study protocol was reviewed and approved by the Ethics Committee of Xuanwu Hospital, Capital Medical University, China. Informed consent was obtained from all the participants and their guardians.

### 2.2 EEG acquisition

Scalp EEGs were recorded using a Bio‐logic and Nihon‐Kohden Neurofax system with a 10–20 electrode placement system at a sampling rate >500 Hz. All children underwent video‐EEG monitoring for 4 h to record awake and sleep periods lasting at least 30 min.

### 2.3 EEG analysis

We randomly selected EEG segments with artifacts free at stage 2 or 3 of NREM sleep for 10 min and an awake state for 5 min. We scored the following: (1) sleep and awake spike rate; (2) location of spikes, especially the frontal EEG focus; and (3) sleep spike ripple rate. We also chose 60 spikes (minimum of 20) in each segment to measure the amplitudes and durations of the prominent negative wave with a cursor and computed their average values.^20^, ^21^

As described previously,^16^, ^17^ we analyzed the EEG data using an average montage. Spikes were marked in the EEG traces using a low frequency of 0.53 Hz (10 s/page), while ripples were identified in the temporal expansion (2 s/page) of the EEG with a low‐cut filter at 80 Hz.^15^, ^18^ Ripples were defined as events of 80–250 Hz with at least four consecutive oscillations standing out in morphology and amplitude from the background in the 80 Hz high‐pass‐filtered signal.^22^ If a ripple occurred 50 ms before or after the spike peak, the event was marked as a spike ripple.^23^, ^24^ One reviewer marked the ripples, which were subsequently checked and discussed with a second reviewer. We counted the moment of the event regardless of the number of channels involved.^16^, ^25^

**FIGURE 1:**
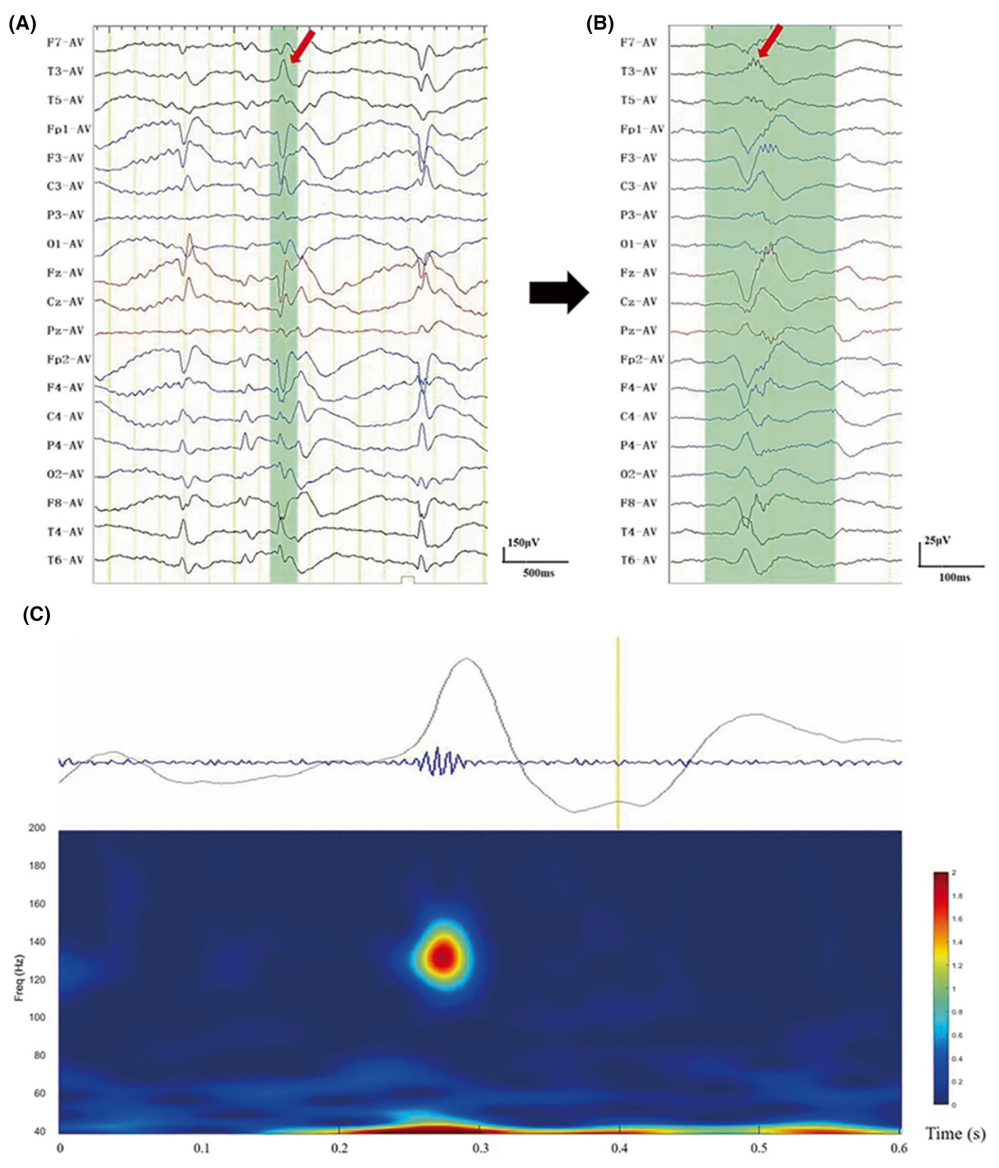
Examples of time‐frequency spectra and the corresponding EEG traces in a patient with atypical benign epilepsy of childhood with centrotemporal spikes. (A) EEG settings were as follows: paper speed, 10 s/page; 30 μV/mm; low frequency (LF), 0.53 Hz; and high frequency (HF), 120 Hz. (B) Filtered EEG corresponds to the green section in (A): paper speed, 2 s/page; 3 μV/mm; LF, 80 Hz; HF, 120 Hz. (C) Time‐frequency analysis for channel T3 in (A) and (B) showed an isolated spectra spot above 80 Hz.

The EEG segments containing visually inspected HFOs were further subjected to time‐frequency analysis using a wavelet transform. On the spectrogram of each inspected HFO, only a “spectral island” in the frequency range of 80–200 Hz was defined as a true HFO^12^, ^15^, ^18^ (Figure 1). The analysis was performed using MATLAB R2020a (Mathworks Inc.).

### 2.4 Statistical analysis

We analyzed the distribution of continuous variables using the Shapiro–Wilk test. Then, to compare the three groups, we used one‐way ANOVA or the Kruskal–Wallis H test, followed by Bonferroni statistical tests to avoid cumulation of type‐I errors. Categorical variables of clinical or EEG data were analyzed using the chi‐squared test. To adjust for confounding factors, the variables having a *p*‐value less than 0.1 were selected in multivariate logistic regression analysis.^26^ A receiver operating characteristic (ROC) curve was used to evaluate the predictive value of the independent predictor, and the results were reported as areas under the curves (AUCs). Moreover, we estimated the correlation of spikes and spike ripples with age and the time to last seizure using partial correlation analysis.

Statistical significance was set at a *p*‐value less than 0.05. All the statistical analyses were performed using SPSS for Windows, version 26.0 (IBM Corp.).

## 3 RESULTS

### 3.1 Demographic and clinical data

Sixty children (33 boys and 27 girls) met the inclusion criteria: 15 with typical seizure‐free BECTS, 26 with active typical BECTS, and 19 with active atypical forms of BECTS. The age at seizure onset was lower in patients with atypical forms of BECTS than in those with typical BECTS (*p* = 0.004). Children in the seizure‐free group were marginally older than those in the active group but the difference was insignificant (*p* = 0.057) (Table 1).

**TABLE 1:** Patient characteristics in three groups.

	Seizure‐free BECTS (*n* = 15)	Active BECTS (*n* = 26)	BECTS with atypical presentations (*n* = 19)	*p‐Value*	p‐Value for post‐hoc tests
Clinical information					
Male (%)	9 (60.0)	17 (65.4)	7 (36.8)	0.164	‐
Age, years	10.73 ± 1.91	9.15 ± 2.17	7.26 ± 1.88	<0.001*	*p*_1_ = 0.057; *p*_2_ = 0.009*
Age at seizure onset, years	7.67 ± 2.38	7.54 ± 2.14	5.47 ± 1.58	0.002*	*p*_1_ = 1.000; *p*_2_ = 0.004*
Family history of epilepsy, *n* (%)	3 (20.0)	6 (23.1)	4 (21.1)	1.000	‐
EEG characteristics					
Frontal focus, *n* (%)	2 (13.3)	5 (19.2)	5 (26.3)	0.715	‐
Sleep spike rate (/min)	11.30 (4.50–57.60)	34.50 (26.33–46.03)	63.30 (51.70–74.60)	<0.001*	*p*_1_ = 0.462; *p*_2_ = 0.001*
Sleep spike amplitude (μV)	70.60 (31.20–109.30)	122.25 (81.48–169.18)	146.10 (115.00–211.00)	0.004*	*p*_1_ = 0.329; *p*_2_ = 0.118
Sleep spike duration (ms)	87.80 (81.10–92.80)	92.65 (85.53–100.55)	98.30 (91.40–103.60)	0.009*	*p*_1_ = 0.425; *p*_2_ = 0.175
Sleep spike ripples	0 (0–1.00)	2.00 (1.00–4.25)	17.00 (8.00–30.00)	<0.001*	*p*_1_ = 0.008*; *p*_2_ < 0.001*
Awake spike rate (/min)	1.00 (0–4.00)	3.00 (1.00–9.50)	15.80 (5.00–34.00)	<0.001*	*p*_1_ = 0.240; *p*_2_ = 0.009*

*Note*: Values are shown as number (%), mean ± SD or median (interquartile range).

Abbreviations: BECTS, benign epilepsy of childhood with centrotemporal spikes; *p*_1_, difference between active and seizure‐free BECTS; *p*_2_, difference between typical and atypical forms of BECTS.

* *p* < 0.05.

Four AEDs were used, including valproate acid (VPA), levetiracetam (LEV), lamotrigine (LTG), and oxcarbazepine (OXC). We found no significant differences in the medicine categories (VPA, *p* = 0.311; LEV, *p* = 0.307; LTG, *p* = 0.501; OXC, *p* = 0.330) or the number of medicine types (*p* = 0.837) among the three groups.

### 3.2 EEG characteristics

#### 3.2.1 Spikes and ripples in three groups

**FIGURE 2:**
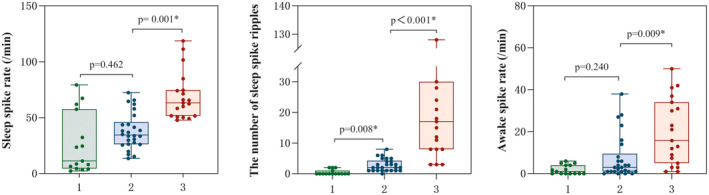
Box plots showing sleep spike rates, the number of sleep spike ripples, and awake spike rates in these three groups. Groups: (1) seizure‐free benign epilepsy of childhood with centrotemporal spikes (BECTS), (2) active BECTS, (3) atypical forms of BECTS during the active phase. The graph shows the total range and the median. *p < 0.05.

There were significant differences among these three groups in the awake spike rate (*p* < 0.001), spike amplitude (*p* = 0.004), spike duration (*p* = 0.009), number of spikes (*p* < 0.001), and spike ripples (*p* < 0.001) during the NREM sleep stages. Moreover, compared with children with seizure‐free BECTS, those with active BECTS had a significantly higher number of spike ripples (*p* = 0.008); however, there was no significant difference in the spike rate during the awake (*p* = 0.240) and sleep (*p* = 0.462) periods. In addition, the awake spike rate (*p* = 0.009), number of spikes (*p* = 0.001), and spike ripples (*p* < 0.001) during the NREM sleep were significantly higher in patients with atypical forms of BECTS than in those with active BECTS (Figure 2). However, there was no significant difference in spike amplitude and duration between seizure‐free and active BECTS or between typical and atypical forms of BECTS (Table 1).

The frontal focus was most common in atypical forms of BECTS, but there was no significant difference in the frequency of frontal focus among different groups (*p* = 0.715).

Multivariate analysis was carried out using the aforementioned variables with *p*‐values less than 0.1. Spike ripple was regarded as an independent predictor of seizure activity and atypical forms of BECTS, with adjusted ORs of 4.714 and 1.455, respectively (Tables 2).

**TABLE 2:** Multivariate logistic regression analyses for prognosis of BECTS.

Variables	Active BECTS		Atypical forms of BECTS	
	*p*	OR (95% CI)	*p*	OR (95% CI)
Age	0.278	‐	0.243	‐
Age at seizure onset	‐	‐	0.310	‐
Sleep spike rate	‐	‐	0.080	‐
Sleep spike ripples	0.003*	4.714 (1.701–13.065)	0.049*	1.455 (1.001–2.113)
Awake spike rate	‐	‐	0.525	‐

Abbreviations: CI, confidence interval; OR, odds ratio.

* *p* < 0.05.

#### 3.2.2 Predictive value: ROC curves

**FIGURE 3:**
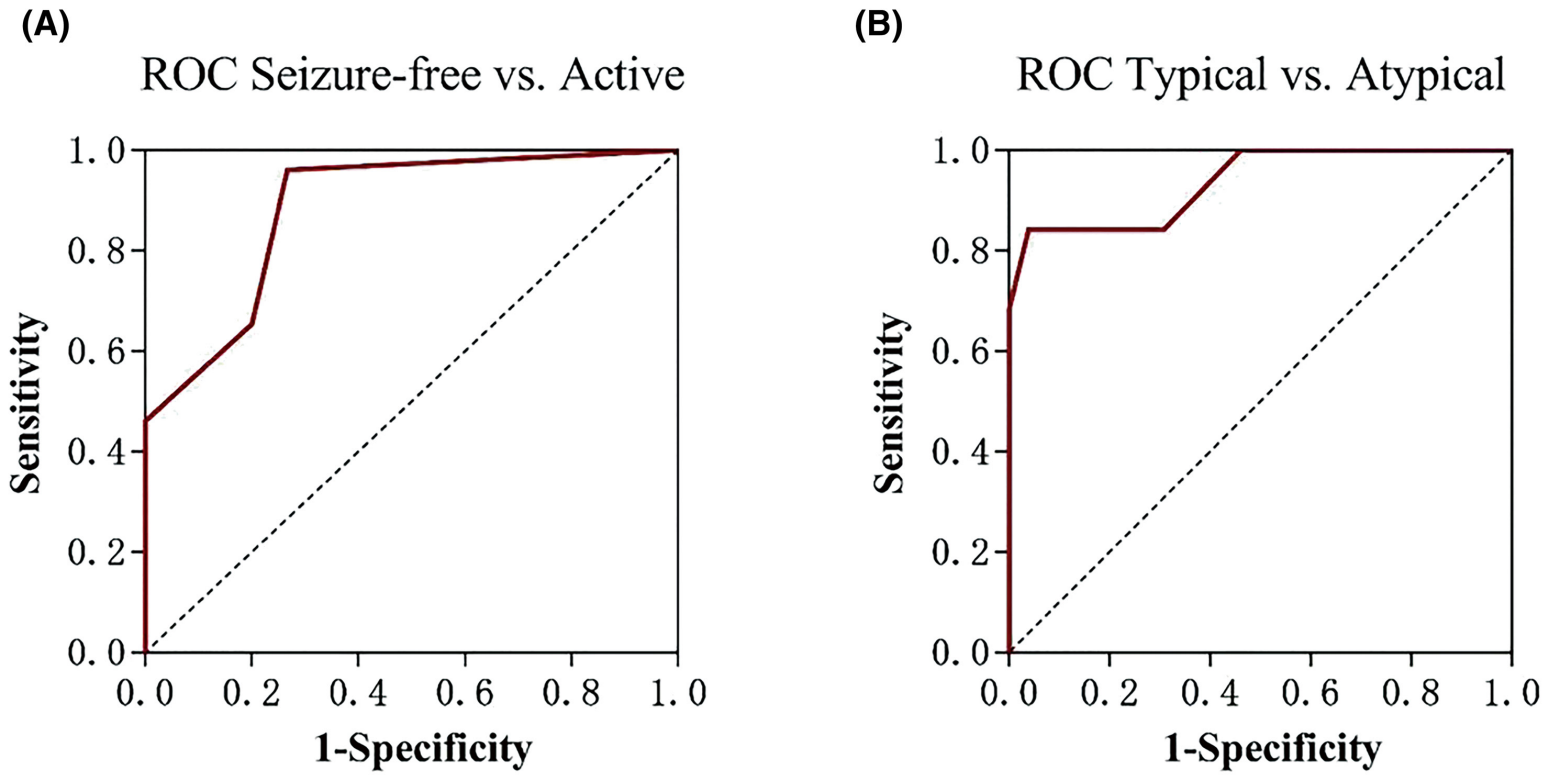
Receiver operating characteristic (ROC) curves of spike ripples to predict the seizure activity (A) and atypical presentations (B).

The ROC for seizure‐free BECTS versus active BECTS showed that the AUC for spike ripples was better than random classification (AUC = 0.885; 95% CI 0.780–0.990, Figure 3A), and a threshold of >0 showed the highest sensitivity (96.15%) and specificity (73.33%).

The ROC curve for atypical forms of BECTS showed that the AUC for spike ripples was better than random classification (AUC = 0.936; 95% CI, 0.865–1.000; Figure 3B). A threshold of >0.6/min spike ripple rate showed the highest sensitivity (84.21%) and specificity (96.15%).

#### 3.2.3 Correlation of spikes and spike ripples with age and the time since the last seizure

The time to the last seizure was longer, and the age of children was older; thus, these factors were included in the partial correlations. In typical BECTS, after controlling for the time since the last seizure, as age increased, the spike ripple rate (*ρ* = −0.379; *p* = 0.016) and the ratio of ripples per spike (*ρ* = −0.331; *p* = 0.037) decreased. After controlling for age, significant correlations were demonstrated between the spike ripple rate (*ρ* = −0.409; *p* = 0.009) or the ratio (*ρ* = −0.464; *p* = 0.003) and the time since the last seizure. But the spike rates did not have a correlation (Table 3).

**TABLE 3:** Partial correlation of spikes and spike ripples with age and the time since the last seizure in BECTS.

	Sleep spike rate	Sleep spike ripple rate	The ratio of ripples per spike
Age	*ρ* = −0.154	*ρ* = −0.379	*ρ* = −0.331
	*p* = 0.342	*p* = 0.016*	*p* = 0.037*
Time since last seizure	*ρ* = −0.104	*ρ* = −0.409	*ρ* = −0.464
	*p* = 0.524	*p* = 0.009*	*p* = 0.003*

* *p* < 0.05.

In atypical forms of BECTS, we did not find a similar correlation between these indicators (Table S1).

## 4 DISCUSSION

Most childhood‐onset BECTS will remit in adolescence, but some children with BECTS may suffer from frequent seizures or atypical presentations over time. Lack of treatment or premature withdrawal of drugs might lead to seizure recurrence and neurocognitive impairment.^13^ However, AEDs may cause adverse reaction.^27^ Thus, it is important to estimate the atypical course and possibility of seizure recurrence in patients with BECTS. In this study, our findings suggest that scalp spike ripple may be not only a predictor for atypical forms of BECTS but also an indicator to reflect the conditions of seizure control and spike ripples may have age‐dependent changes.

### 4.1 Typical and atypical forms of BECTS

Previous studies^16^, ^28^, ^29^ found that the age at seizure onset was younger, and the rates of sleep and waking spikes were higher in children with atypical forms of BECTS than in those with typical forms of BECTS; the results are consistent with our findings. Moreover, several studies have confirmed the influence of interictal epileptic discharges^29^, ^30^; along with the increase in spikes, the number of motor seizures and oral dysfunction would increase. Spikes reflect large excitatory postsynaptic potentials mediated by the abnormally hypersynchronous neurons firing which can generate positive symptoms.^30^, ^31^ And they immediately result in prolonged membrane hyperpolarization mediated by recurrent postsynaptic inhibition which is related to slow waves and explain the presence of negative myoclonus.^30^, ^32^ Moreover, a long period of discharge could lead to developmental dysfunction of the cortex, which might explain the neurocognitive and behavioral impairment in patients with atypical forms of BECTS.^29^ In addition, Kanemura et al.^29^, ^33^, ^34^ suggested that in atypical BECTS, the frontal focus was sustained for an extended period in sleep EEGs associated with brain maturation. We discovered a higher proportion of frontal focus in children with atypical forms of BECTS, but no significant difference, probably due to the lack of follow‐up.

Spike ripple in scalp sleep EEGs was regarded as a factor for distinguishing between typical and atypical forms of BECTS. In 2016, van Klink et al.^16^ recruited eight patients with typical rolandic epilepsy and eight patients with atypical or symptomatic rolandic epilepsy to analyze their EEGs. They found that the presence of more than five spike ripples in EEGs of 10 min was a predictor for atypical or symptomatic epilepsy. The EEGs they analyzed included those during awake or sleep periods; notably, spikes and ripples were activated during sleep.^2^, ^35^ In our investigations, we included a larger sample size of children with typical and atypical forms of BECTS and analyzed the data using a multivariate logistic regression model to exclude the disturbance of confounding factors. We found that spike ripple rates >0.6/min might indicate atypical presentations. Based on these findings, we speculate that the disturbance of pathological HFOs to immature brain networks might change the advanced functions of the brain and affect the cognitive functions of children with atypical forms of BECTS.^12^

Childhood epilepsy with centrotemporal spikes is a clinical spectrum consisting of syndromes with various severities. Previous studies^8^, ^12^ confirmed that HFOs were more prominent in CSWS than in BECTS. Based on the findings and our present findings, we speculate that the number of HFOs might increase gradually from BECTS to atypical BECTS and CSWS. In the early course, it is difficult to distinguish typical and atypical forms of BECTS using traditional EEGs. To a certain extent, comparing the rate of spikes between the two entities mentioned above is unreliable, but the spike ripple is a more accurate indicator.

### 4.2 Seizure activity of BECTS

Previous cohort studies^36^, ^37^, ^38^ confirmed that seizure recurrence of BECTS was associated with higher spike rates in the awake or sleep period and more frontal focus sustained for a long period. Our present findings indicated similar results, but the difference was not statistically significant. Interictal epileptic discharge has been demonstrated to be the primary biomarker for predicting remission and recurrence of BECTS, but its accuracy is low.^6^ Interestingly, according to earlier researches, spike rates in BECTS always increase in the 6 and 12 months after onset and then decrease over time,^37^ which might explain the weak correlation between seizure activity and the number of spikes.^17^ Therefore, when a patient experiences an improvement in seizures, the sleep spike rates might increase.

The spike ripple rate is an independent indicator of seizure control. The relationship between HFOs and disease activity has long been discussed in studies. In the last century, scholars explored the relationship in kainic rats and confirmed that the earlier the HFO occurrence, the shorter the latent period of seizure occurrence.^39^ A study of intracranial recordings later found that HFOs were more sensitive to AEDs reduction than spikes.^9^ In 2016, a new exploration of scalp HFOs emerged, and Qian et al.^15^ demonstrated that when treating atypical benign partial epilepsy with corticosteroids, the decrease in spike ripple was more obvious than that in the spike. Subsequently, Kramer et al.^17^ analyzed 10 patients with active BECTS and 13 patients with seizure‐free BECTS; they concluded that the spike ripple could be used to distinguish between these two groups, but the spike could not. Based on a larger sample, we removed the disturbance of confusing factors and found that scalp spike ripple was an independent predictor of seizure risk. We demonstrated that the presence of spike ripples might indicate active BECTS, with a sensitivity of 96.15% and specificity of 73.33%. Previous finding^16^ showed that children with rolandic spikes but without epilepsy usually had less than two ripples in the EEGs of 10 min, which was slightly different with our results, probably because these children the study recruited had developmental problems. HFOs might reflect pathologically interconnected clusters of principal neurons discharged in common.^40^ Thus, spikes co‐occurring with ripples are more closely related to epileptogenesis and may provide greater reliability for predicting active BECTS.

In addition, Kobayashi et al.^18^ and Kramer et al.^17^ showed that in patients with BECTS, the time since the last seizure was shorter in those with more spike‐related ripples than in those with spikes alone. We reached a similar conclusion and confirmed the strong relationship between spike ripples and disease activity. Moreover, we also found that spike ripples rather than spikes decreased with age in patients with BECTS; the finding is consistent with that in the study by Ohuchi et al.^8^ The ratio of ripples per spike signifies the proportion of ripples superimposed on each spike. The decreased ratio demonstrates that epileptogenesis diminished with age, which explains the age dependence of this disease. However, we did not observe the aforementioned results for atypical forms of BECTS, probably because seizure‐free patients with atypical presentations were not enrolled in the study. Thus, a larger patient cohort is needed to verify this finding.

The significance of HFOs is different with the spikes. Spikes are thought to represent synchronous postsynaptic potentials derived by hyperexcitable neurons,^31^ whereas HFOs might reflect synchronized co‐firing of principal neural clusters.^40^ The brain regions that generate spikes and HFOs have spatial overlap, which means these two events share similar hyperactive neuronal networks.^41^ The cortex generating HFOs was, however, smaller than that generating spikes.^42^ With regard to HFOs, a considerably larger area of the synchronized cortex is required for the corresponding activity to be visible on the scalp.^22^ Therefore, occasional synchronization of HFOs in a relatively broad cortex may form a pattern appearing on the scalp, which possibly explains the higher rate of spikes.^41^

Physiological and pathological HFOs coexist in scalp EEGs. Mooij et al.^43^ and Chu et al.^44^ reported the presence of ripples in EEGs without spikes and suggested that the ripples were physiological. Therefore, previous studies on scalp ripples in patients with epilepsy focused on the ripple overlapping the spike,^16^, ^17^ which is associated with epileptogenic properties.^8^ In other words, it is reasonable to primarily analyze ripples co‐occurring with spikes.

In this study, most of the patients recruited had taken AEDs. We did not find a significant difference in the use of AEDs between the three groups. Therefore, medication might not sufficiently explain the differences in spikes and ripples among the groups. Certainly, in the future, we could design a prospective study to distinguish the drug effect from the remission of the disease.

Nevertheless, our study has some limitations. Prior research have discussed the effects of development and aging on HFOs. Somatosensory evoked HFOs were enhanced in both healthy children and aged subjects.^45^, ^46^ However, the relationship between age and scalp HFOs in epileptic patients is still controversial.^43^, ^47^ Future studies could include more patients with various types of epilepsy to explore the changes in physiological and pathological scalp HFOs with age.

## 5 CONCLUSION

Considering the variation in the severity of BECTS and the potential adverse reactions to AEDs, it is difficult for clinicians to determine whether to use AEDs and when to withdraw the drugs. This study indicates that spike ripple is a non‐invasive biomarker for estimating the risk of seizure recurrence and atypical presentations. The present findings would help prevent unnecessary or insufficient treatment and assist drug screening.

## AUTHOR CONTRIBUTIONS

Wei Sun, Yichen Ji designed the study. Jun Zhang, Hongjuan Lu, Haoran Yang, Xuan Zhang, Huixin Liu, Wei Zhou, and Xiaoling Zhang collected the data. Yichen Ji and Wenjian Liu analyzed the data. Yichen Ji drafted the manuscript. Wei Sun revised the manuscript.

## FUNDING INFORMATION

This research was supported by the Beijing Hospitals Authority Clinical Medicine Development of special funding (grant number: XMLX202117) and the National Natural Science Foundation of China (grant number: 81571267).

## CONFLICT OF INTEREST STATEMENT

None declared.

## Supporting information


Table S1

## Data Availability

The data are not publicly available due to privacy restrictions. The data that support the findings of this study are available from the corresponding author upon reasonable request.
