# Supplementary material for: Correlation between scalp high‐frequency oscillations and prognosis in patients with benign epilepsy of childhood with centrotemporal spikes
Source: CNS Neurosci Ther. 2023 May 8;29(10):3053–61. doi: 10.1111/cns.14246 (PMC10493670; doi:10.1111/cns.14246)
Supplement: Supplementary file 1 — Table S1 [file CNS-29-3053-s001.docx]

**Supplementary Table**. Partial correlation of spikes and spike ripples with age and the time since the last seizure in atypical forms of BECTS.

|  | Sleep spike rate | Sleep spike ripple rate | The ratio of ripples per spike |
| --- | --- | --- | --- |
| Age | ρ=0.092  p=0.716 | ρ=0.213  p=0.396 | ρ=0.188  p=0.455 |
| Time since the last seizure | ρ=0.054  p=0.831 | ρ=-0.189  p=0.454 | ρ=-0.194  p=0.440 |
